# 

**Published:** 1856-12

**Authors:** 


### CONTENTS.

ORIGINAL COMMUNICATIONS— PAGE.

Report on the Changes in the Composition and Properties of the Milk of the Human Female, produced by Menstruation and Pregnancy. By N. S. Davis, M.D. .......... 535

Case of Compound Fracture of the Skull, with Symptoms of Compression—Operation. By Wm. Manson, M.D. and John T. Richardson, M.D. .......... 547

SELECTIONS, ..........

A Case of Obstinate Regurgitation of the Contents of the Stomach, Treated by the Inhalation of Chloroform; with Remarks on Regurgitation. By Isaac E. Taylor, M.D. .......... 551

BOOK NOTICES, ..........

The Obstetric Memoirs and Contributions of James Y. Simpson, M.D. 564

Materia Medica, or Pharmacology and Therapeutics. By Wm. Tully, M.D. .......... 665

American Journal of Pharmacy. Edited by Wm. Proctor, .......... 565

On the Diseases of Infants and Children. By Fleetwood Churchill, M.D. 566

A Treatise on Therapeutics and Pharmacology. By George B. Wood, M.D. .......... 567

MEDICAL INTELLIGENCE, ..........

Proceedings of the Annual Meeting of the Æsculapian Society, .......... 568

EDITORIAL, ..........

Rush Medical College, .......... 573

Number of Medical Journals, .......... 573

To Subscribers, .......... 574

Medical Ethics, .......... 574

